# Supplementary material for: Expression of FGFR1–4 in Malignant Pleural Mesothelioma Tissue and Corresponding Cell Lines and its Relationship to Patient Survival and FGFR Inhibitor Sensitivity
Source: Cells. 2019 Sep 16;8(9):1091. doi: 10.3390/cells8091091 (PMC6769772; doi:10.3390/cells8091091)
Supplement: Supplementary file 1 [file cells-08-01091-s001.pdf]

Table S1: Correlation of FGFR1 staining pattern with clinical and pathology data.

		FGFR1						
		**0**		**1**		**2**		**p**
		**n**	**%**	**n**	**%**	**n**	**%**	
Age	< 60	0	0	13	32	9	25	ns
	≥ 60	4	100	28	68	27	75	
Sex	female	0	0	12	29	10	28	ns
	male	4	100	29	71	26	72	
Histology	non-epithelioid	3	75	9	22	10	28	ns
	epithelioid	1	25	32	78	26	72	
Stage	early	0	0	15	37	13	36	ns
	late	4	100	26	63	23	64	
Treatment overview	BSC	0	0	7	17	8	22	ns
	CHT	1	25	13	32	19	53	
	CHT+RT	1	25	1	2	1	3	
	CHT+S	1	25	8	20	2	6	
	TMT	1	25	12	29	6	17	

BSC: best supportive care, CHT: chemotherapy, RT: radiotherapy, S: surgery, TMT: trimodality therapy, ns: not significant

Table S2: Correlation of FGFR3 staining pattern with clinical and pathology data.

		FGFR3						
		**0**		**1**		**2**		**p**
		**n**	**%**	**n**	**%**	**n**	**%**	
Age	< 60	16	34	5	16	1	50	ns
	≥ 60	31	66	27	84	1	50	
Sex	female	14	30	7	22	1	50	ns
	male	33	70	25	78	1	50	
Histology	non-epithelioid	12	26	10	31	0	0	ns
	epithelioid	35	74	22	69	2	100	
Stage	early	13	28	15	47	0	0	ns
	late	34	72	17	53	2	100	
Treatment overview	BSC	6	13	9	28	0	0	ns
	CHT	16	34	16	50	1	50	
	CHT+RT	2	4	1	3	0	0	
	CHT+S	8	17	2	6	1	50	
	TMT	15	32	4	13	0	0	

BSC: best supportive care, CHT: chemotherapy, RT: radiotherapy, S: surgery, TMT: trimodality therapy, ns: not significant

Table S3: Correlation of FGFR4 staining pattern with clinical and pathology data.

		FGFR4				
		0		1		p
		n	%	n	%	
Age	< 60	19	25	3	50	ns
	≥ 60	56	75	3	50	
Sex	female	18	24	4	67	**0.024**
	male	57	76	2	33	
Histology	non-epithelioid	20	27	2	33	ns
	epithelioid	55	73	4	67	
Stage	early	27	36	1	17	ns
	late	48	64	5	83	
Treatment overview	BSC	15	20	0	0	ns
	CHT	31	41	2	33	
	CHT+RT	2	3	1	17	
	CHT+S	9	12	2	33	
	TMT	18	24	1	17	

BSC: best supportive care, CHT: chemotherapy, RT: radiotherapy, S: surgery, TMT: trimodality therapy, ns: not significant

Table S4: Sensitivity of the MPM cell lines to BLU9931.

Cell line	Histology	IC_{50} (µM) BLU9931
Meso49	bi	60.7
Meso62	sarc	95.8
Meso84	sarc	39.6
Meso92	bi	6.2
Meso161	bi	7.9
Meso189	epi	24.1
Meso194	epi	20.7
Meso205	epi	24.1
Meso208	epi	2.9
Meso221	epi	6.3
VMC28	epi	n.i.
VMC40	bi	3.0
VMC45	epi	n.i.

epi: epithelioid, bi: biphasic, sarc: sarcomatoid, n.i.: no inhibition

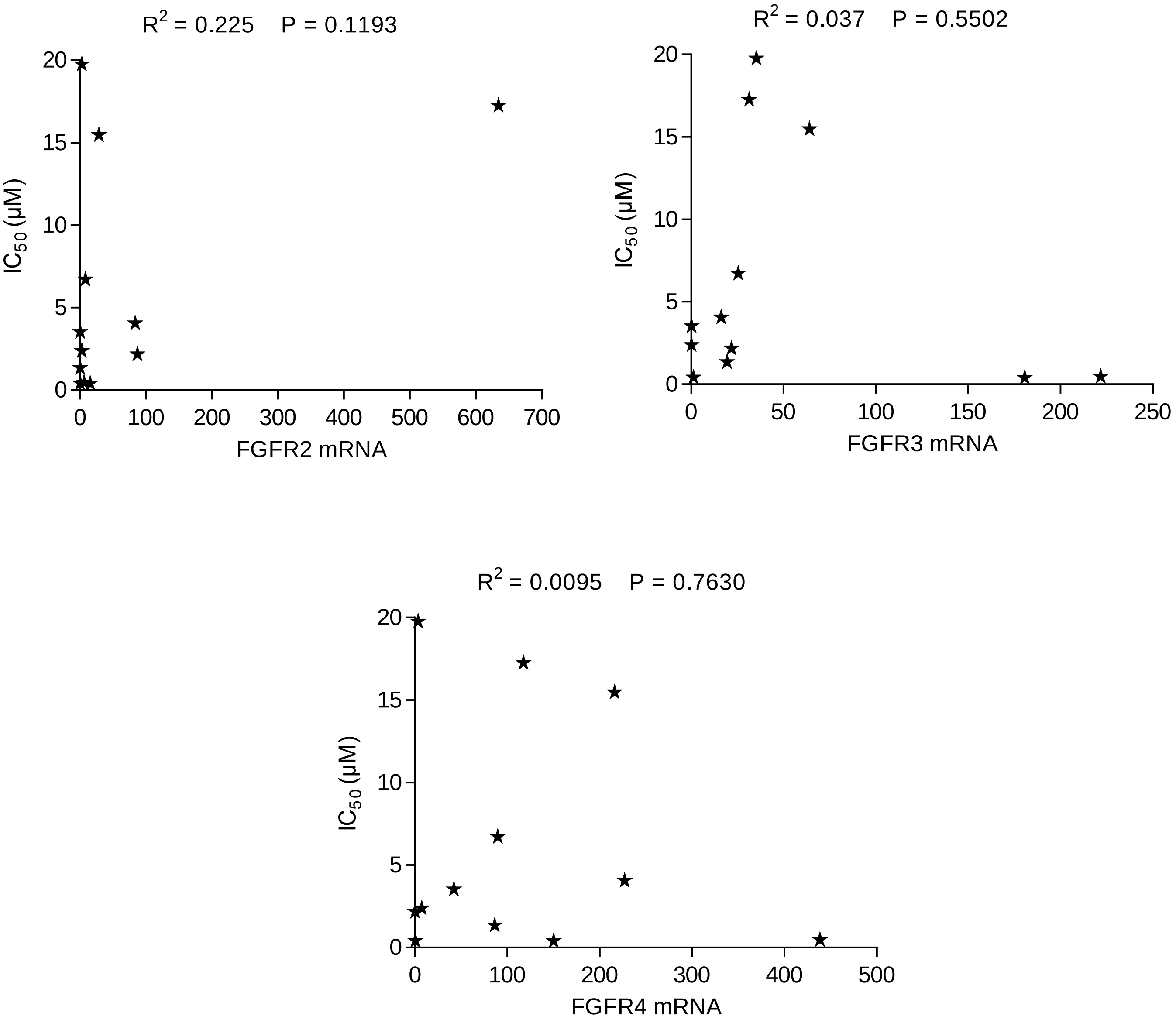

Figure S1: Correlation analysis of infigratinib sensitivity with FGFR mRNA expression levels. Infigratinib IC_{50} values were plotted as function of FGFR2 (upper left panel), FGFR3 (upper right panel) and FGFR4 (lower panel) mRNA expression levels.

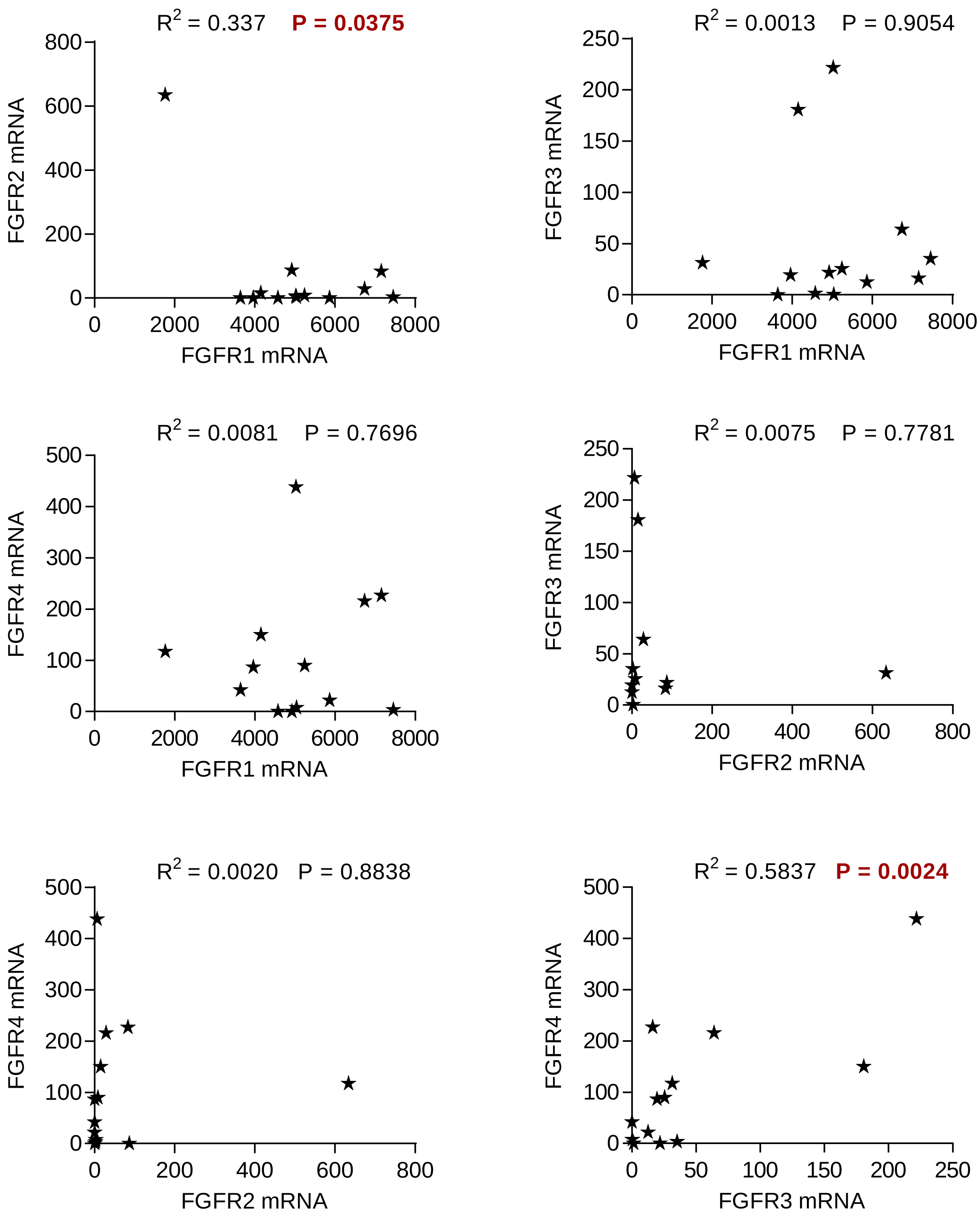

Figure S2: Correlation analysis of FGFR mRNA expression levels. Messenger RNA levels of FGFR1-FGFR4 were correlated in pairwise comparisons.
